# Effectiveness of Preventive Therapy for Persons Exposed at Home to Drug-Resistant Tuberculosis, Karachi, Pakistan

**DOI:** 10.3201/eid2703.203916

**Published:** 2021-03

**Authors:** Amyn A. Malik, Neel R. Gandhi, Timothy L. Lash, Lisa M. Cranmer, Saad B. Omer, Junaid F. Ahmed, Sara Siddiqui, Farhana Amanullah, Aamir J. Khan, Salmaan Keshavjee, Hamidah Hussain, Mercedes C. Becerra

**Affiliations:** Emory University Rollins School of Public Health, Atlanta, Georgia, USA (A.A. Malik, N.R. Gandhi, T.L. Lash);; Yale University, New Haven, Connecticut, USA (A.A. Malik, S.B. Omer);; Global Health Directorate, Indus Health Network, Karachi, Pakistan (A.A. Malik, J.F. Ahmed, S. Siddiqui);; Interactive Research and Development, Singapore (A.A. Malik, A.J. Khan, H. Hussain);; Emory University School of Medicine, Atlanta (N.R. Gandhi, L.M. Cranmer);; Emory + Children’s Pediatric Institute, Atlanta (L.M. Cranmer);; The Indus Hospital, Karachi (F. Amanullah);; Harvard University, Cambridge, Massachusetts, USA (A.J. Khan, S. Keshavjee, M.C. Becerra);; Partners In Health, Boston, Massachusetts, USA (S. Keshavjee, M.C. Becerra);; Brigham and Women’s Hospital, Boston (S. Keshavjee, M.C. Becerra)

**Keywords:** drug-resistant tuberculosis infection, contact investigation, preventive therapy, fluoroquinolone, prophylaxis, latent infection, tuberculosis and other mycobacteria, Karachi, Pakistan, antimicrobial resistance, bacteria

## Abstract

In Karachi, Pakistan, a South Asian megacity with a high prevalence of tuberculosis (TB) and low HIV prevalence, we assessed the effectiveness of fluoroquinolone-based preventive therapy for drug-resistant (DR) TB exposure. During February 2016–March 2017, high-risk household contacts of DR TB patients began a 6-month course of preventive therapy with a fluoroquinolone-based, 2-drug regimen. We assessed effectiveness in this cohort by comparing the rate and risk for TB disease over 2 years to the rates and risks reported in the literature. Of 172 participants, TB occurred in 2 persons over 336 person-years of observation. TB disease incidence rate observed in the cohort was 6.0/1,000 person-years. The incidence rate ratio ranged from 0.29 (95% CI 0.04–1.3) to 0.50 (95% CI 0.06–2.8), with a pooled estimate of 0.35 (95% CI 0.14–0.87). Overall, fluoroquinolone-based preventive therapy for DR TB exposure reduced risk for TB disease by 65%.

## Abstract 2

Fluoroquinolone-based preventive therapy reduced risk for tuberculosis disease by 65%.

Tuberculosis (TB) is the leading infectious cause of death globally and the 9th leading cause overall (*1*). TB causes ≈10 million new cases and 1.7 million deaths annually (*1*). Annually, ≈650,000 TB patients have multidrug-resistant (MDR) TB, defined as TB that is resistant to both isoniazid and rifampin (*1*). Treatment for MDR TB is toxic, complex, and prolonged, and it has a success rate of only 55% (*1*–*3*). Therefore, preventive interventions, including preventive therapy and future vaccines, are essential to reduce cases and deaths from MDR TB (*4*,*5*).

Delivering effective treatment for exposure to drug-resistant (DR) TB is central to the work of Zero TB Initiative coalitions, which aim to rapidly drive down TB rates worldwide (*6*). Household contacts of persons with DR TB are at high risk for TB (*7*) and are prime candidates for preventive interventions (*8*). Available standard preventive therapies are not expected to protect persons exposed to MDR TB because the infecting TB strain in the exposed person is highly likely to be resistant to isoniazid and rifampin. A meta-analysis of 33 studies found that >80% of household contacts of persons with DR TB in whom TB occurred also had isoniazid-resistant strains (*9*). Thus, household contacts of persons with DR TB should receive treatment under the assumption that they, too, are infected with a DR *Mycobacterium tuberculosis* strain (*9*).

Evidence is limited regarding effective preventive regimens for MDR TB, in contrast to the abundant evidence available for preventive therapy in isoniazid-sensitive TB (*10*). Although data from randomized controlled trials are not available to guide the approach to preventive therapy for MDR TB, observational studies from the Federated States of Micronesia, United States, United Kingdom, and South Africa have shown efficacy of fluoroquinolone-based preventive therapy in adults and children (*11*–*17*). The largest observational study with a comparison arm, from the Federated States of Micronesia, described 104 household contacts of persons with MDR TB who received preventive therapy with a fluoroquinolone-based regimen for 12 months. During 3 years of follow-up, TB did not occur in any of the contacts who received preventive therapy; in 3 (20%) of the 15 contacts who refused treatment, MDR TB occurred. A meta-analysis of observational studies determined MDR TB preventive therapy to be 90% effective, and a wide range of 9%–99% effectiveness was reported (*18*).

Most studies of preventive therapy for MDR TB have been conducted in either high-resource settings or settings with a high prevalence of HIV. Hence, evaluations of the effectiveness of MDR TB preventive therapy in other settings are needed. In Karachi, Pakistan, which has a high TB burden and low HIV prevalence setting (annual TB incidence of 265/100,000 and HIV prevalence [among persons 15–49 years of age] of 0.1%) (*1*,*19*), we examined the effectiveness of fluoroquinolone-based 2-drug preventive therapy for high-risk household contacts of persons with DR TB.

## Methods

### Setting, Study Design, and Population

During February 2016–March 2017, we prospectively enrolled household contacts of 100 consecutive (index) patients beginning treatment for culture-confirmed DR TB at the Indus Hospital in Karachi, Pakistan. Because this study was conducted in a programmatic setting, we identified index patients with any DR TB, not only the subset of patients with MDR TB. Household contacts of index patients whose isolates were shown in drug-susceptibility testing to be resistant to a fluoroquinolone in addition to other first-line drugs but not resistant to any of the second-line injectables were eligible for the study. Of the 100 index patients, 97 had documented resistance to rifampin; 15 also had documented resistance to a fluoroquinolone. Full details of the cohort are reported elsewhere (*20*,*21*).

The study cohort consisted of all children and adults residing with index patients at the time of the diagnosis of DR TB. At the baseline evaluation, all household contacts were evaluated for TB clinically, including chest radiograph and sputum testing if they were able to produce sputum. We conducted HIV testing if the person had HIV risk factors or if the index patient had HIV co-infection. We excluded household members already receiving treatment for TB at the time of this evaluation (n = 8) or those in whom TB was diagnosed in the clinical evaluation (i.e., co-prevalent TB patients [n = 3]). We offered preventive therapy with a fluoroquinolone-based 2-drug regimen for 6 months to remaining household contacts who met these criteria: 0–4 years of age; 5–17 years of age with a positive tuberculin skin test (TST) result or evidence of immunocompromising condition, such as diabetes, HIV, or malnutrition (body mass index [BMI] <18.5 kg/m^2^); or >18 years of age with evidence of an immunocompromising condition, such as diabetes, HIV, or malnutrition (BMI <18.5 kg/m^2^). Persons who did not meet these criteria were not prescribed preventive therapy but were followed for the occurrence of active TB disease.

We provided 1 of 4 preventive regimens, each consisting of 2 drugs for a duration of 6 months (Table 1). Moxifloxacin-based regimens were given to household contacts of index patients with a levofloxacin-resistant TB strain. Ethambutol was the companion drug of choice unless it was not available in the correct dosing form; in that case, ethionamide was used.

**Table 1:** Preventive therapy regimens in study of persons exposed at home to drug-resistant tuberculosis, Karachi, Pakistan, February 2016–March 2017*

Regimen	Drug 1, dose	Drug 2, dose
Levofloxacin/ethambutol	Levofloxacin, <5 y: 15–20 mg/kg, >5 y: 7.5–10 mg/kg; max. dose 1,000 mg/d	Ethambutol, 15–25 mg/kg; max. dose 2,000 mg/kg
Levofloxacin/ethionamide	Levofloxacin, <5 y: 15–20 mg/kg, >5 y: 7.5–10 mg/kg; max. dose 1,000 mg/d	Ethionamide, 15–20 mg/kg; max. dose 750 mg/kg
Moxifloxacin/ethambutol	Moxifloxacin, 7.5–10 mg/kg; max. dose 400 mg/d	Ethambutol, 15–25 mg/kg; max. dose 2,000 mg/kg
Moxifloxacin/ethionamide	Moxifloxacin, 7.5–10 mg/kg; max. dose 400 mg/d	Ethionamide, 15–20 mg/kg; max. dose 750 mg/kg
*Max., maximum.		

A study physician clinically evaluated persons on preventive therapy every 2 months for 6 months. Between clinic visits, a study worker visited each household monthly to monitor for occurrence of TB symptoms or adverse events and to assess treatment adherence. Treatment adherence was self-reported and cross-checked through pill counts during home visits. We conducted follow-up on persons who completed the 6-month preventive regimen every 2 months at home or by telephone to monitor for occurrence of TB symptoms. We conducted follow-up symptom screening on persons who did not receive preventive therapy every 2 months at home or by telephone to monitor for occurrence of TB symptoms until the end of the study period. Any household contact experiencing TB symptoms was referred to The Indus Hospital clinic for further evaluation, including chest radiography and sputum testing if able to produce sputum.

### Analysis

The primary outcome of interest was the effectiveness of preventive therapy in household contacts, defined as disease-free survival 2 years after the diagnosis of DR TB in the index patient. To establish an historical untreated group for comparison, we searched the published literature to find systematic reviews and meta-analyses of studies of the incidence of TB disease in close contacts after exposure to a person with TB. We found 2 such studies (*7*,*22*). We then searched for studies that were conducted after these meta-analyses were published. We did not restrict the search to studies that evaluated TB incidence only in persons exposed to drug-resistant TB disease, because no difference is expected in transmissibility or progression on the basis of drug-resistance profile (*7*,*23*). We used the definition of an incident case of TB disease and TST positivity as defined by each study.

From the identified studies, we extracted the incidence of TB disease among untreated household contacts by age, year postexposure, TST-positive results or high-risk classification, and preventive therapy status, if provided (*22*,*24*–*29*) (Table 2).

**Table 2:** Details of studies from which data were extracted for analysis in study of persons exposed at home to drug-resistant tuberculosis, Karachi, Pakistan, February 2016–March 2017*

Characteristic	Becerra et al. (*25*)	Fox et al. (*22*)	Reichler et al. (*26*)	Martin-Sanchez et al. (*27*)	Sloot et al. (*28*)	Saunders et al. (*29*)
Setting	Peru	Global	US and Canada	Spain	Netherlands	Peru
Year	2013	2013	2019	2019	2014	2017
HHC age group, y						
<15	1,299	N/A	879	77	1,489	NA
>15	3,411	N/A	3,611	876	7,757	1,910
IR or risk	IR and risk	IR and risk	IR and risk	IR and risk	Risk	Risk
IR or risk by PT status	No PT for DR TB exposure	No	Yes	Yes	Yes	No
IR or risk by age and year of follow-up	Yes	Not by age but by year of follow-up	No	No cases in children	No	No
IR or risk by risk group	No	No	Yes	Yes	No	Yes
IR or risk reported	<15 y, Y 1: 2,079/100,000 p-y; <15 y, Y 2: 315/100,000 p-y; >15 y, Y 1: 2,610/100,000 p-y; >15 y, Y 2: 1,309/100,000 p-y; risk: 163/4,515 (3.6%)	Y 1: 1,478/100,000 p-y; Y 2: 831/100,000 p-y; risk: 898/65,935 (1.4%)	Rate: 951/100,000 p-y; 5 y risk for TST-positive contacts without PT: 49/446 (11.0%)	Rate: 1970/100,000 p-y; 5 y risk for TST-positive contacts not completing PT: 6/72 (8.3%)	2 y risk in TST-positive contacts without PT: 9/372 (2.4%)	2.5 y risk for medium- to high-risk contacts in validation cohort: 57/1,335 (4.3%)
Other limitations	Some children received isoniazid-based PT	NA	P-y accumulated over 5 y	No cases in children less than 15 y; p-y accumulated over 5.3 y	Definition of incidence >6 mo	HHCs >15 y

*DR TB, drug-resistant tuberculosis; HHC, household contacts; IR, incidence rate; PT, preventive therapy; p-y, person-years; TST, tuberculin skin test; Y, year of follow-up; NA, data not available.

We calculated the observed incidence rate of TB disease in contacts who received preventive therapy by dividing the number of persons in whom TB occurred by the person-years accumulated by the cohort over 2 years. Cumulative incidence of TB over 2 years was calculated by dividing the number of persons in whom TB occurred by the total number of persons who received preventive therapy. We applied the incidence rates extracted from the literature (Table 2) to our cohort to calculate the expected number of persons in whom TB disease would have occurred within 2 years of exposure to a person with DR TB in the absence of preventive therapy. We calculated the expected incidence rate by dividing the expected number of persons in whom TB disease would have occurred by the total person-years accumulated in our cohort over 2 years. To assess the effectiveness of preventive therapy, we then compared the expected incidence rate and cumulative incidence of TB from the studies in Table 2 with the observed incidence rate and cumulative incidence in our cohort. Incidence rate ratio (IRR), risk ratio (RR), incidence rate difference (IRD), and risk difference were used for comparison, depending on the available data. We calculated the number needed to treat to prevent 1 case of TB as the total number of persons receiving preventive therapy divided by the number of TB cases averted. Number of TB cases averted was calculated by subtracting the observed number of TB cases from the expected number of TB cases.

We generated pooled estimates of IRR and RR by using inverse-variance weighting with random effects for the effectiveness of preventive therapy that are robust to a range of different assumptions. We evaluated the validity of the pooled estimation method from the random effects model by a simulation study with 10,000 replications using a Poisson distribution for the incidence rate and a binomial distribution for risk for each study. Data were analyzed by using Stata version 15 (StataCorp, https://www.stata.com) and SAS software version 9.4 (SAS Institute, https://www.sas.com). This study was approved by the Institutional Review Boards of Interactive Research and Development, Harvard Medical School, and Emory University.

## Results

Of the 800 household members enrolled in the study, 8 were receiving treatment for TB disease at the time of the baseline evaluation. Of the 792 remaining persons, we verbally asked 737 (93.1%) about symptoms; 402 (54.5%) met criteria for further evaluation, and we evaluated 326 (81.1%), none of which were infected with HIV. Active TB disease was diagnosed in 3 (0.9%) persons. Of the remaining 323 persons, 215 met the study criteria and were offered preventive therapy; within that cohort, median age was 7 years (interquartile range [IQR] 3–16) and median BMI was 14.8 kg/m^2^ (IQR 13.4–16.9); 52% persons were male. Of the persons offered preventive therapy, 172 accepted and contributed 336 person-years of observation; 7 of these participants were household contacts of patients with rifampin-susceptible strains of TB. Preventive therapy was declined by 43 of 215 persons who were eligible for treatment, but they remained under observation. The 43 persons who did not start treatment were older (median age 16 years [IQR 3–22]) than those who started preventive treatment (median age 7 years [IQR 3–15]). The 2 groups had no other notable differences (Table 3). Of the whole cohort (91% [n=157] of those who began preventive therapy), 82% (n = 654) completed 2 years of observation, and 70% (n = 121) of those who started treatment completed it. There were no deaths during the follow-up period.

**Table 3:** Demographics and clinical characteristics of household contacts exposed to drug-resistant tuberculosis free of disease at baseline in study of preventive therapy in Karachi, Pakistan, February 2016–March 2017*

Characteristic	No. (%) or median [IQR]			
	Total, n = 789†	On PT, n = 172	Did not start PT, n = 43	Not eligible for PT, n = 574
Age group, y	19 [10–32]	7 [3–15]	16 [3–22]	24 [15–36]
<15	283 (36)	128 (74)	21 (49)	134 (23)
>15	506 (64)	44 (26)	22 (51)	440 (77)
Sex				
M	423 (54)	91 (53)	20 (47)	312 (54)
F	366 (46)	81 (47)	23 (53)	262 (46)
BMI, kg/m^2^	18.1 [14.8–24.0], n = 616	14.8 [13.4–16.9], n = 171	15.2 [13.4–16.9], n = 42	21.6 [17.1–26.0], n = 403
Presence of symptoms	n = 737	n = 172	n = 43	n = 522
Cough, duration	10 (1)	3 (2)	2 (5)	5 (1)
Fever	7 (1)	1 (1)	3 (7)	3 (1)
Weight loss	12 (2)	1 (1)	2 (5)	9 (2)
Additional TB risk factors	n = 737	n = 172	n = 43	n = 522
History of TB	9 (1)	0 (0)	0 (0)	9 (2)
TST >5 mm	6/136 (4)	6/64 (9)	0/11 (0)	0/61 (0)
Index patient resistant to FQ	138 (19)	16 (9)	11 (26)	111 (21)
Regimen given				
Levofloxacin/ethambutol	NA	102 (59)	NA	NA
Levofloxacin/ethionamide	NA	54 (31)	NA	NA
Moxifloxacin/ethambutol	NA	11 (6)	NA	NA
Moxifloxacin/ethionamide	NA	5 (3)	NA	NA
TB disease occurred during follow-up	2 (0.3)	2 (1)	0	0
*FQ, fluoroquinolone; NA, not applicable; PT, preventive therapy; TB, tuberculosis; TST, tuberculin skin test. †Excluding 3 contacts found to have TB and 8 already on treatment for TB at time of screening.				

We calculated the expected incidence of TB disease in the group that received preventive therapy in Karachi by using incidence rates stratified by age and year of observation from a DR TB household cohort from Peru (*24*,*25*). Had no preventive therapy been given, we would have expected TB disease to occur in 4.7 patients, on the basis of the 336 person-years accumulated by our cohort (incidence rate 14/1,000 person-years). Only 2 patients in our study had TB over the 2 years of observation, resulting in a TB incidence rate of 6.0/1,000 person-years and cumulative incidence of 1.2%. Both case-patients had received preventive therapy (Appendix Table 1). IRR comparing observed and expected number of TB cases was 0.40 (95% CI 0.05–2.0) and IRD was −8.0/1,000 person-years (95% CI –23 to 7.1). Number needed to treat to avert 1 TB case was 64.

**Figure 1:**
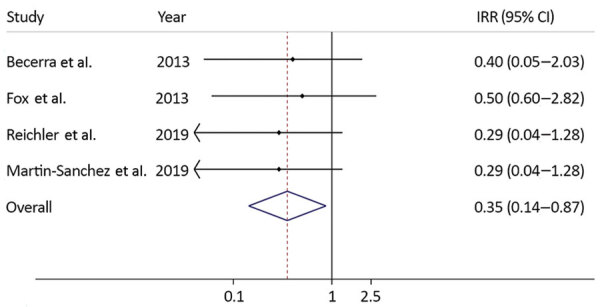
Incidence rate ratios for effectiveness of preventive therapy using data from published studies and a summary measure in study of preventive therapy for persons exposed at home to drug-resistant tuberculosis, Karachi, Pakistan, February 2016–March 2017. Solid line on y axis indicates null. Dotted line indicates pooled estimate of preventive therapy effectiveness. Blue diamond indicates 95% CI. Small diamonds indicate point estimates of preventive therapy effectiveness using data from each study with its CI. IRR, incident rate ratio.

We performed the same exercise by using TB incidence rates from 2 other studies and a meta-analysis to demonstrate the potential range of IRR and IRD (*22*,*26*,*27*) (Table 4). Equivalent results were achieved by using rates from Reichler et al. (*26*) and Martin-Sanchez et al. (*27*); the expected number of TB cases was 6.6 and IRR was 0.29 (95% CI 0.04–1.3). By using rates of TB disease incidence in household contacts of TB patients as determined by Fox et al. (*22*), we calculated the IRR to be 0.50 (95% CI 0.06–2.8). The pooled estimate for IRR was 0.35 (95% CI 0.14–0.87) (Figure 1). Using the simulation study, the median IRR was 0.42 (2.5th–97.5th percentile 0.18–0.79).

**Table 4:** Incidence rate comparison of effectiveness of preventive therapy for tuberculosis in published studies in study of persons exposed at home to drug-resistant tuberculosis, Karachi, Pakistan*

Characteristic	Becerra et al. (*25*)	Fox et al. (*22*)	Reichler et al. (*26*)	Martin-Sanchez et al. (*27*)
No. expected cases	4.7	3.9	6.6	6.6
Expected IR per 1,000 p-y	15	12	20	20
IRR (95% CI)	0.40 (0.05–2.0)	0.50 (0.06–2.8)	0.29 (0.04–1.3)	0.29 (0.04–1.3)
IR difference per 1,000 p-y (95% CI)	−8.0 (–23.0 to 7.1)	−5.7 (–20.0 to 8.5)	−14 (–31.0 to 3.4)	−14 (–31.0 to 3.4)
NNT	64	91	37	37
Preventive fraction in exposed, %	57.5	48.7	69.5	69.7

*IR, incidence rate; IRR, incidence rate ratio; NNT, number needed to treat; p-y, person-years.

**Figure 2:**
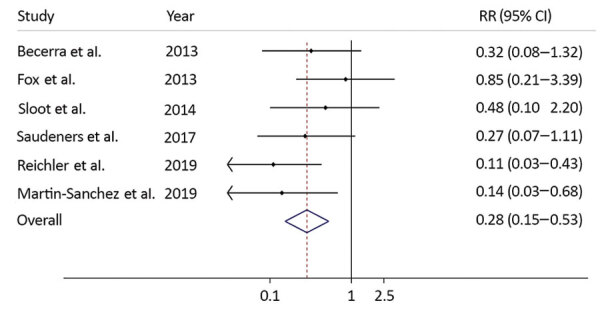
Risk ratios for effectiveness of preventive therapy using data from published studies and a summary measure in study of preventive therapy for persons exposed at home to drug-resistant tuberculosis, Karachi, Pakistan, February 2016–March 2017. Solid line on y axis indicates null. Dotted line indicates pooled estimate of preventive therapy effectiveness. Blue diamond indicates 95% CI. Small diamonds indicate point estimates of preventive therapy effectiveness using data from each study with its CI. RR, risk ratio.

We found 6 studies that estimated the risk for TB disease in household contacts exposed to a TB patient in the absence of preventive therapy, including the 4 studies we used for incidence rate calculations (*22*,*24*–*29*). By using risk figures from these 6 studies, we estimated the pooled RR to be 0.28 (95% CI 0.15–0.53) (Figure 2; Appendix Table 2). By using the simulation study, we calculated the median RR to be 0.36 (2.5th–97.5th percentile 0.17–0.68).

When we applied an alternative definition of an incident TB case, in which diagnosis occurred earlier (>30 days as opposed to >180 days after diagnosis in the index patient), and used data from Sloot et al. (*28*) as a sensitivity analysis, the estimated RR for preventive therapy was 0.11 (95% CI 0.03–0.44). Using this figure in the pooled analysis resulted in an estimated pooled RR of 0.22 (95% CI 0.12–0.42).

## Discussion

In our cohort of 172 DR TB household contacts who received fluoroquinolone-based preventive therapy, we observed 2 patients with TB disease over the course of 2 years. Applying the rates observed in a cohort of DR TB households from Lima, Peru (*25*), we would have expected to observe almost 5 TB cases over the same period. Thus, by providing preventive therapy, we averted almost 3 TB cases resulting in an effectiveness rate of 60%.

Household contacts are a combination of several populations with different risk levels and biologic susceptibility. Saunders et al. (*29*), in a study from Peru, created a risk score to predict the persons in whom TB would occur after at-home exposure to TB; they demonstrated that 90% of TB cases occurred among persons at high or medium risk over 10 years. In that study, 2 of the risk factors for TB were low BMI and age; the score predicted the risk for TB independent of TB-infection status (*29*). Other studies have also documented increased risk for TB in children <5 years of age and persons with low BMI. We provided preventive therapy to household members at known high risk for TB on the basis of demographics and clinical manifestation; 35% of those started on preventive therapy were <5 years of age. Hence, the 60% effectiveness rate is likely an underestimate of its true effectiveness because the rates we used to calculate expected TB cases came from the whole household cohort and not only from persons at highest risk for incident TB. Some of the children in the comparison cohort also received isoniazid-based preventive therapy, which might have lowered their risk for TB.

By using TB incidence rates from 2 studies from the United States and Spain and a meta-analysis by Fox et al. (*22*), we calculated a range of 2–5 TB cases averted through this program and an effectiveness of 50%–71%. The meta-analysis also included persons who were prescribed preventive therapy, and it did not differentiate between those at higher and lower risk for incident TB disease, which probably resulted in lower overall incidence rate. The other 2 studies measured TB incidence rate over 5 years of follow-up, but the highest risk for incident TB disease is within the first 2 years after exposure. Thus, applying the rate measured over 5 years to a cohort followed for 2 years might result in underestimation of expected number of incident TB cases. The pooled estimate of effectiveness of the preventive therapy in this Karachi cohort compared with all 4 studies was 65%.

By using the pooled relative risk, we estimated the effectiveness of preventive therapy to be 72%. This estimate is comparable to the effectiveness that we found by using incidence rate data from other studies (*22*,*25*–*27*) and gives more confidence in interpretation of our results. Of note, these studies also had some of the limitations highlighted previously. Our results are also consistent with the TB risk reduction reported with use of isoniazid-based preventive therapy for drug-susceptible TB (relative risk 0.40, 95% CI 0.31–0.52) (*30*). For MDR TB, Marks et al. (*18*), in their meta-analysis of published observational studies on preventive therapy for MDR TB exposure, estimated a risk reduction of 90% (range 9%–99%).

A key limitation of our study was reliance on at-home symptom screening for diagnosis of incident TB and on household members to report TB diagnoses or initiation of treatment for TB during the study period. The parent study was designed to evaluate operational feasibility of providing preventive therapy and was not designed as an effectiveness study, which explains these design features. This limitation could have led to an ascertainment bias. We do not, however, expect that our estimates would be substantially biased with this approach. In the same population during 2008–2011, Amanullah et al. (*31*) conducted a household cohort study by using a similar approach and found a high TB incidence of 5.4% among children in the first year after exposure to a person with DR TB. Our use of rates from countries with low to moderate TB burden, such as Peru, for comparison with the rates from this study in Pakistan, a country with a high burden of TB, might also have biased our results, potentially underestimating the protective effect of preventive therapy. Furthermore, the use of 5-year risks from some of the comparison studies might have overestimated the effectiveness of preventive therapy, given that in our study we calculated cumulative incidence at 2 years. This possibility is not very likely because, in those studies, most of the TB cases occurred within the first 2 years. 

Strengths of our study include the prospective design, which resulted in >91% retention at 2 years and a high completion rate of preventive therapy. Our results were robust to a range of different assumptions and showed a similar decrease in TB incidence after provision of preventive therapy to that demonstrated in other observational studies.

In summary, in a setting with high TB burden and low HIV prevalence, we found that a fluoroquinolone-based, 2-drug preventive therapy reduced the risk for TB disease in high-risk persons exposed at home to DR TB by 65%. This study adds to the growing evidence base for effectiveness of preventive therapy for DR TB and MDR TB and is consistent with evidence that a fluoroquinolone-based 2-drug regimen can be used to protect children and adults exposed at home to DR *M. tuberculosis* strains.

Appendix Additional information about preventive therapy for persons exposed at home to drug-resistant tuberculosis, Karachi, Pakistan.
