# Supplementary material for: Effectiveness of Preventive Therapy for Persons Exposed at Home to Drug-Resistant Tuberculosis, Karachi, Pakistan
Source: Emerg Infect Dis. 2021 Mar;27(3):805–12. doi: 10.3201/eid2703.203916 (PMC7920671; doi:10.3201/eid2703.203916)
Supplement: Appendix — Additional information about preventive therapy for persons exposed at home to drug-resistant tuberculosis, Karachi, Pakistan. [file 20-3916-Techapp-s1.pdf]

Article DOI: https://doi.org/10.3201/eid2703.203916

Effectiveness of Preventive Therapy for Persons Exposed at Home to Drug-Resistant Tuberculosis, Karachi, Pakistan

Appendix

Appendix Table 1. Characteristics of household contacts in whom incident tuberculosis occurred in study of preventive therapy for persons exposed at home to drug-resistant tuberculosis, Karachi, Pakistan*

No.	Age	Sex	Time on PT	Regimen	No. household members on PT	Time to TB development	Type of TB	TB Tx outcome	Comments
1	19 y	F	5 mo	Levofloxacin 750 mg and ethionamide 500 mg	5	16 mo	RR-TB	Lost to follow up	Variable compliance with PT because of adverse events. 3 members of household had TB disease.
2	15 y	F	6 mo	Levofloxacin 750 mg and ethionamide 500 mg	6	19 mo	DS-TB (culture negative)	Completed treatment	2 members of the family on concurrent TB treatment and failing treatment.

*DS-TB, drug-susceptible tuberculosis; PT, preventive therapy; RR-TB, rifampin-resistant tuberculosis; TB, tuberculosis; Tx, treatment.

Appendix Table 2. Risk comparison of effectiveness of tuberculosis preventive therapy in published studies, in study of persons exposed at home to drug-resistant tuberculosis*

Characteristic	Becerra et al. 2013 (*1*)	Fox et al. 2013 (*2*)	Reichler et al. 2019 (*3*)	Martin-Sanchez et al. 2019 (*4*)	Sloot et al. 2014 (*5*)	Saunders et al. 2017 (*6*)
No. of expected cases	6.2	2.3	18.9	14.3	4.2	7.3
Expected risk	3.6% at 2 y	1.4% at 2 y	11.0% at 5 y	8.3% at 5 y	2.4% at 2 y	4.3% at 2.5 y
RR (95% CI)	0.32 (0.08–1.3)	0.85 (0.21–3.4)	0.11 (0.03–0.43)	0.14 (0.03–0.68)	0.48 (0.10–2.2)	0.27 (0.07–1.1)
Risk diff per 100 persons (95% CI)	−2.5 (–4.1 to –0.76)	−0.2 (–1.8 to 1.4)	−9.8 (–13.1 to –6.5)	−7.2 (–13.8 to –0.59)	−1.3 (–3.5 to –0.98)	−3.1 (–5.0 to –1.2)
NNT	41	573	11	14	78	32
Preventive fraction in exposed	67.8%	14.6%	89.4%	86.1%	51.9%	72.8%

*NNT, number needed to treat; RR, risk ratio.

References

1. Becerra MC, Franke MF, Appleton SC, Joseph JK, Bayona J, Atwood SS, et al. Tuberculosis in children exposed at home to multidrug-resistant tuberculosis. Pediatr Infect Dis J. 2013;32:115–9. PubMed https://doi.org/10.1097/INF.0b013e31826f6063

2. Fox GJ, Barry SE, Britton WJ, Marks GB. Contact investigation for tuberculosis: a systematic review and meta-analysis. Eur Respir J. 2013;41:140–56. PubMed

3. Reichler MR, Khan A, Sterling TR, Zhao H, Chen B, Yuan Y, et al.; Tuberculosis Epidemiologic Studies Consortium Task Order 2 Team. Tuberculosis Epidemiologic Studies Consortium Task Order 2 Team. Risk factors for tuberculosis and effect of preventive therapy among close contacts of persons with infectious tuberculosis. Clin Infect Dis. 2020;70:1562–72. PubMed https://doi.org/10.1093/cid/ciz438

4. Martin-Sanchez M, Brugueras S, de Andrés A, Simon P, Gorrindo P, Ros M, et al.; Contact Tracing Group of the Tuberculosis Investigation Unit of Barcelona. Tuberculosis incidence among infected contacts detected through contact tracing of smear-positive patients. PLoS One. 2019;14:e0215322. PubMed https://doi.org/10.1371/journal.pone.0215322

5. Sloot R, Schim van der Loeff MF, Kouw PM, Borgdorff MW. Risk of tuberculosis after recent exposure. A 10-year follow-up study of contacts in Amsterdam. Am J Respir Crit Care Med. 2014;190:1044–52. PubMed

6. Saunders MJ, Wingfield T, Tovar MA, Baldwin MR, Datta S, Zevallos K, et al. A score to predict and stratify risk of tuberculosis in adult contacts of tuberculosis index cases: a prospective derivation and external validation cohort study. Lancet Infect Dis. 2017;17:1190–9. PubMed
